# Supplementary material for: Depression and Complicated Grief, and Associated Factors, of Bereaved Family Members of Patients Who Died of Malignant Pleural Mesothelioma in Japan
Source: J Clin Med. 2022 Jun 13;11(12):3380. doi: 10.3390/jcm11123380 (PMC9225633; doi:10.3390/jcm11123380)
Supplement: Supplementary file 1 [file jcm-11-03380-s001.zip › jcm-1744012-supplementary.pdf]

Supplementary Table S1. Bivariate analysis of PHQ-9 and BQG scores with clinical social factors.

	p value	
	Depression	Complicated grief
Male patient *	0.715	0.401
Patient's age at diagnosis **	0.515	0.912
Survival **	0.786	0.006
Bereaved family member's age	0.506	0.106
Female bereaved family member *	0.951	0.297
Spouse bereaved family member *	0.209	0.133
Time of bereavement **	0.893	0.157
Financial impact of patient's MPM on family **	0.880	0.040
Anger against asbestos **	0.001	0.001
Compensated by workmen's accident compensation insurance *	0.590	0.573
Compensated by asbestos-related health damage relief system *	0.136	0.178
Received surgery *	0.195	0.123
Received chemotherapy *	0.956	0.384
Received radiotherapy *	0.502	0.814
Received palliative care *	0.987	0.403
Experienced end-of-life discussion with patient*	0.678	0.902
Satisfactied with care on diagnosis *	0.297	0.020
Satisfied with care when the patient became critical *	0.297	0.013
Patient died at home *	0.962	0.148
Patient died sooner than expected *	0.426	0.728
GDI Score**	0.272	0.000
CES Score**	0.991	0.089

* t-test

**Correlation analysis
